# Dynamical Analysis of Universal Masking on the Pandemic

**DOI:** 10.3390/ijerph18179027

**Published:** 2021-08-27

**Authors:** Brandon Kaiheng Tay, Carvalho Andrea Roby, Jodi Wenjiang Wu, Da Yang Tan

**Affiliations:** Science, Mathematics and Technology, Singapore University of Technology and Design, 8 Somapah Road, Singapore 487372, Singapore; brandontay6@gmail.com (B.K.T.); andwea3@gmail.com (C.A.R.); jodiwwj@gmail.com (J.W.W.)

**Keywords:** SEIR model, SEIS model, mask wearing, compartmental model, epidemic modelling

## Abstract

We investigate the impact of the delay in compulsory mask wearing on the spread of COVID-19 in the community, set in the Singapore context. By using modified SEIR-based compartmental models, we focus on macroscopic population-level analysis of the relationships between the delay in compulsory mask wearing and the maximum infection, through a series of scenario-based analysis. Our analysis suggests that collective masking can meaningfully reduce the transmission of COVID-19 in the community, but only if implemented within a critical time window of approximately before 80–100 days delay after the first infection is detected, coupled with strict enforcement to ensure compliance throughout the duration. We also identify a delay threshold of about 100 days that results in masking enforcement having little significant impact on the Maximum Infected Values. The results therefore highlight the necessity for rapid implementation of compulsory mask wearing to curb the spread of the pandemic.

## 1. Introduction

In the light of the COVID-19 pandemic, the Singapore government declared with effect as of 14 April 2020, an enforcement of compulsory mask wearing in public spaces, 89 days after the first case of COVID-19 was detected in Singapore.

The usage of masks is known to effectively decrease the infection rate. It has shown success in limiting community spread of SARS 2003 [1], and more recently, in Taiwan’s management of COVID-19 [2]. Recent hypothetical studies on masking by the states of New York and Washington suggests a potential prevention of up to 45% of their projected death rates [3]. In this study of potential face-mask usage for the general public, the authors investigated how public masking can control the infection, in the context of the USA. However, not much was mentioned about how the delayed enforcement of such a policy would affect the infection numbers. In another study on public masking [4], the authors studied how factors such as the filtering capability of different mask materials, as well as sociological behaviour patterns on masking, would affect the efficacy of this policy. A comprehensive data-driven study on COVID-19 waves worldwide and strategies to mitigate them conducted by Lai and Cheong [5] also did not provide detailed analysis of mask wearing policies and its effectiveness. Concluding this literature review, it is apparent that in-depth studies on the impact of delayed mask enforcement on the spread of COVID-19 in the community is limited.

This paper thus seeks to investigate the relationship between delay in compulsory mask wearing and the Maximum Infected Values, through a series of scenario-based what-if analysis. We would be considering 3 scenarios using 2 compartmental models—the SEIR and the SEIRS model. The use of compartmental models for modelling the spread of COVID-19 has been explored recently, such as to study the lockdown measures [6,7], or with variant to include the effects of age-structure within the population [8] and different social settings [9]. For the SEIR model, we consider a complete compliance, and gradual noncompliance over time. Using an SEIRS model, we consider a third scenario taking into account time-limited immunity. Results from simulating these 3 scenarios would hopefully shed light on the effects of delaying the usage of masks and how the containment of epidemic could have been more effective in Singapore.

To model the pandemic from a macroscopic view point, we use modified versions of the model, which consists of a number of compartments described by a system of differential equations in Section 2 and Section 3. To solve these equations, we implemented the model in Python using the Odeint package. We then modify the Delay of Mask Enforcement by plotting several data sets and recording the resulting Maximum Infection Value predicted by our model. This was performed over 3 different scenarios:

- Scenario 1: The first scenario considers the most basic case of complete compliance of the masking enforcement, from the day it was enforced throughout its duration. It also does not account for time-limited immunity [10], where infected individuals become susceptible again after a period of time.
- Scenario 2: The second scenario closely resembles the first, but with the addition of gradual noncompliance of the masking enforcement. The root of noncompliance stems from either lack of medical knowledge, wishful thinking that the pandemic will magically disappear, selfish behaviour of individuals, pandemic fatigue etc. Here, we assume that onset of the noncompliance is triggered by an event, for instance, changes in government policies. As an illustration, the Singapore government announced the Phase 2 of its gradual reopening plan on 18 June 2020 (154 days after the first case in Singapore) where members of the public were allowed to visit shopping malls and dine-in at food establishments, it was observed that a minority of individuals did not comply with the masking regulations.
- Scenario 3: The third scenario is almost identical to the first, but now accounts for time-limited immunity. After 90 days, it has been shown that a recovered individual may become susceptible to the virus again [11]. This scenario serves as a comparison to investigate if the immunity factor would worsen the effects of the delay of mask enforcement on the maximum infected values.

Here we highlight that effective control of the COVID19 pandemic cannot be achieved by the universal masking enforcement alone, but a strong combination factors such as social distancing enforcement, rapid roll-out of mass testing, lockdowns and extensive quarantine measures. However, these are outside the scope of our analysis, as we aim to both model and investigate how the delay of public masking enforcement affects its effectiveness in controlling the pandemic. We have thus considered the effects of universal masking in isolation of the other factors for the purpose of this analysis.

Section 2 and Section 3 of this article describe and explain the SEIR and SEIRS mathematical models we implemented. Section 4 describes the mathematical modelling of the relationship between the mask wearing and the infection rate with respect to time t. Section 5 describes the epidemiological parameters implemented in our models. Brief justification of their values are also included. Section 6 comprises the results and discussion, and is divided into 3 sub-sections. Section 6.1 explains the dynamics of the delay in compulsory mask wearing, and discusses its effects as demonstrated by our models. Section 6.2 explains the effects of this delay on the Maximum Infected Values for each of the 3 scenarios. Section 6.3 discusses the limitations of our study. Finally, Section 7 wraps up the discussion with a conclusion.

## 2. SEIR Model

Compartmentalized mathematical models have been regularly used to model infectious diseases. The most basic SIR (Susceptible, Infected, Removed) was developed in the early twentieth century by Ronald Ross, William Hamer, and many others [12], and has been heavily modified since, with the addition of new compartments and parameters. More recently, the use of similar models have resurfaced to model the COVID19 situation in many countries. Recent epidemiological studies on China [13], Italy [14], India [15] for example, have been conducted with modified versions of such a model. For our study, we used the SEIR [16] and SEIRS [17] variant of the model, as described in Section 2 and Section 3.

The SEIR model consists of several compartments, namely Susceptible, Exposed, Infected and Removed. We expand the model by further differentiating the Removed compartment into 2 new Recovered and Death compartments. The flow between the 5 components are described by the following 5 differential equations:

(1) $$\frac{dS(t)}{dt}=-\beta I\left(t\right)\frac{S(t)}{N}$$

(2) $$\frac{dE(t)}{dt}=\beta I\left(t\right)\frac{S(t)}{N}-\delta E\left(t\right)$$

(3) $$\frac{dI(t)}{dt}=\delta E\left(t\right)-\left(1-\alpha \right)\gamma I\left(t\right)-\alpha \rho I\left(t\right)$$

(4) $$\frac{dR(t)}{dt}=\left(1-\alpha \right)\gamma I\left(t\right)$$

(5) $$\frac{dD(t)}{dt}=\alpha \rho I\left(t\right)$$

where *N* is the total population, S(t),E(t),I(t),R(t) and D(t), are the number of people susceptible, exposed, infected, recovered and dead on day *t*. β is the expected number of people an infected person infects per day, γ is the proportion of recovery per day, δ is the incubation period, α is the fatality rate due to the infection and ρ is the inverse of the average number of days for an infected person to die if he does not recover.

## 3. SEIRS Model

The SEIRS model is similar to the SEIR model such that it has five components as well—Susceptible, Exposed, Infected, Recovered and Death. However, the SEIRS model takes time-limited immunity into account, whereby recovered individuals are prone to becoming susceptible to the disease again after a period of time.

In the SEIRS model, Equations (1) and (4) are then modified to

(6) $$\frac{dS(t)}{dt}=-\beta I\left(t\right)\frac{S(t)}{N}+\epsilon R\left(t\right)$$

(7) $$\frac{dR(t)}{dt}=\left(1-\alpha \right)\gamma I\left(t\right)-\epsilon R\left(t\right)$$

where ϵ is the rate at which a recovered person becomes susceptible again.

## 4. Time-Based Model for Compulsory Mask Wearing

**Figure 1:**
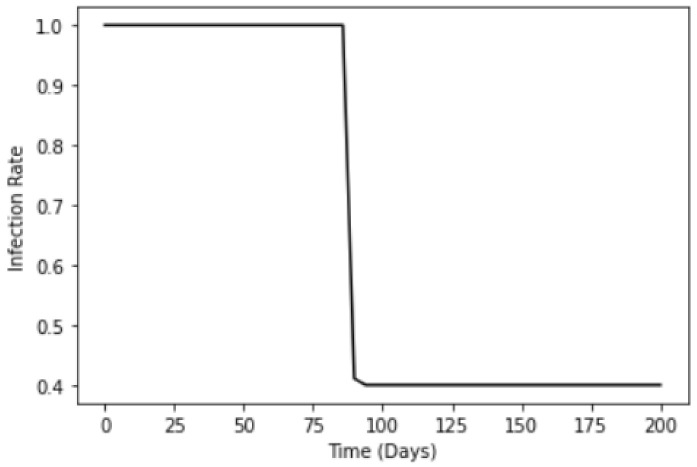
Logistic function to model the infection rate due to compulsory mask wearing m(t) for Scenarios 1 and 3.

Masking is found to decrease the infection rate I(t) by reducing the transmission of respiratory droplets between individuals, which in turn reduces the number of individuals an infected person can infect. Kai et al. [18] showed that the infection rate can be reduced by 60% when universal masking is enforced. There have been other studies that reflect varying rates of infection rate reduction, such as 47% (estimate between 15% and 75%) in Germany [19] and 75% in Arizona during the 2020 summer surge [20], along with numerous others. The disparity of the figures are likely to be due to the influence of other policies such as enforced social distancing and lockdowns. For the purpose of our study, we felt 60% reduction in infection rate was a reasonable value, without being too optimistic or pessimistic.We thus model our infection rate as time-dependent β→β(t) and a function of m(t):

(8) β(t)=κm(t)

where κ is an arbitrary constant and

(9) $$m\left(t\right)=\frac{\beta_{s}-\beta_{c}}{1+e^{-k(-t+t_{0})}}+\beta_{c}$$

where a modified logistic function is used to model this transition by setting the infection before (i.e., at the start of the outbreak) and after the full compliance masking enforcement to be $\beta_{s}=1$ and $\beta_{c}=0.4$ respectively. $t_{0}$ is the number of days after the first case where masking wearing is enforced. Figure 1 shows an example of such logistic function, where for the case of Singapore, we set $t_{0}=89$, since the policy of compulsory mask wearing was implemented 89 days after the first case was uncovered. This model is used in both Scenarios 1 and 3.

To model the gradual noncompliance of universal masking, we make a modification to Equation (9).

(10) $$m\left(t\right)=\frac{\beta_{s}-\beta_{c}}{1+e^{-k_{1}\left(-t+t_{0}\right)}}+\frac{\beta_{c}-\beta_{nc}}{1+e^{-k_{2}\left(-t+t_{1}\right)}}+\beta_{nc}$$

**Figure 2:**
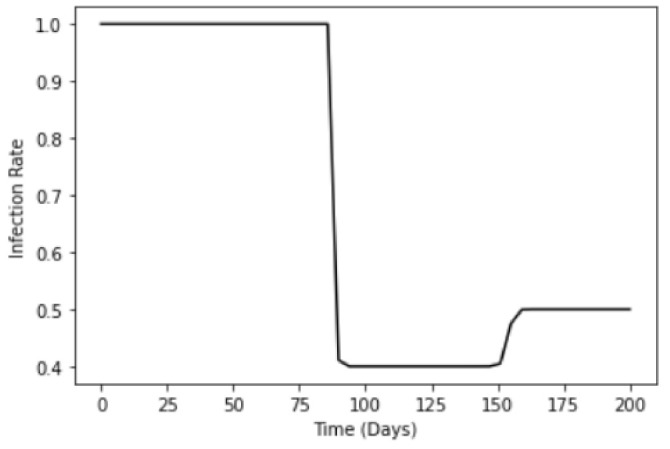
Logistic function to model the infection rate due to compulsory mask wearing m(t) for Scenario 2, where one takes into account the noncompliance.

As individuals began to engage in noncompliance out of complacency and pandemic fatigue, the infection rate, $\beta_{nc}$, would thus increase slightly. Furthermore, compared to quenching of the infection rate due to enforcement of the compulsory mask wearing, the noncompliance will be gradual. This results in a gentler gradient as the infection rate transits from $\beta_{c}\to \beta_{nc}$. The gradients are tuned by the arbitrary constants $k_{1}$ and $k_{2}$, where $k_{1}>k_{2}$. We further assume that the population are able to maintain compliance for a period of time before onset of noncompliance at $t_{1}$, which may be triggered by an event, for example a change in the government’s policy. Figure 2 illustrates one such example, where we have $\beta_{nc}=0.5$ and $t_{1}=154$, taken in context to Singapore’s shift from a full lockdown to gradual resumption of everyday activities 154 days after the first case. The infection rate $\beta_{nc}$ is expected to be lower than $\beta_{s}$ as even with the complacency and fatigue, as there is now a greater situational awareness of the severity and the population in general will take a more cautious outlook compared to pre-pandemic days.

## 5. Epidemiological Parameters

To conduct our analysis in the Singapore context, we estimate the epidemiological parameters described in the earlier sections for the Singapore’s context:

- γ=1/11: According to the position statement released by the National Centre of Disease Control Singapore [21], a person remains infectious for up to 11 days after first contracting COVID-19.
- δ=1/5: The SARS-CoV2 virus has an incubation period of about 5 days [22].
- α=0.000064: Fatality rate is defined as the percentage of deaths among all previously infected individuals. At the time this work was conducted, the number of deaths in Singapore was 26, and the total number of Recovered and Dead compartments was 40,625.
- ρ=1/9: As this number varies greatly across different demographics and is highly unpredictable, we are unable to obtain a proper average. Moreover, owing to the low numbers of COVID-19 deaths in Singapore, it would be inaccurate to calculate an average using this small sample size. Thus, for analysis purposes, we set it at 9 days.

## 6. Results and Discussion

### 6.1. Dynamics of Delay of Compulsory Mask Wearing

**Figure 3:**
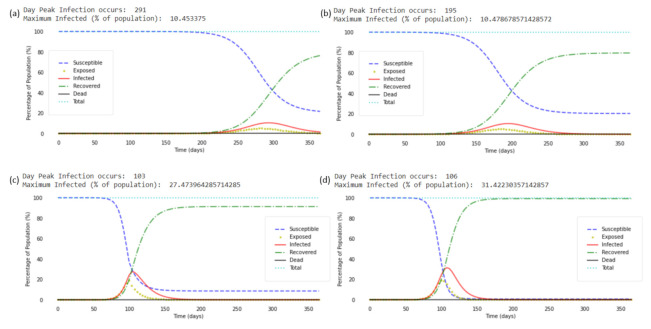
SEIR Plots for delays of (**a**) 0 days, (**b**) 50 days, (**c**) 100 days. (**d**) corresponds to the control case where mask wearing is not enforced.

Upon applying the SEIR models to the 3 scenarios described in the above sections, we are able to obtain Maximum Infected Values by simulating different values of delay in mask enforcement. Considering the most basic case of Scenario 1; where there is complete compliance throughout the duration of mask enforcement, and the absence of time-limited immunity, Figure 3 shows the cases where mask wearing were to be enforced (a) on the day the first case of COVID-19 was detected in Singapore; (b) after a 50 days delay; (c) after a 100 days delay; and (d) not enforced at all.

From Figure 3, it can be deduced that earlier enforcement of mask wearing both reduces the Maximum Infected Value as well as increases the number of days taken to reach the Maximum Infected Value. This is reflected by right-ward shift of the maxima of the Infected compartment, as well as a lower maxima value of the infected curve. The infected number increases until it reaches a global maxima, before decreasing as predicted by compartmental models of such form. This global maxima is termed as the Maximum Infected Value. For the case of (a) 0 days of delay in mask enforcement, the maximum infected value is approximately 10.453%, and the peak occurs at day 291 after the first case was detected. For the case of a (b) 50 days delay, the maximum infected value is approximately 10.479%, and the peak occurs on day 195. For the case of a (c) 100 days delay, the maximum infected value is much higher, at approximately 27.474%, and the peak occurs much earlier, on day 103. This is very close to the case where (d) no public masking is enforced, where the maximum infected value is approximately 31.422% and the peak occurs on day 106. From a policy point of view, this suggests that early universal masking would indeed be an effective control, not only to reduce the infection, but more critically to flatten the curve so as to not overwhelm the medical resources.

### 6.2. Effects of Delay of Mask Enforcement on Maximum Infected Value

**Figure 4:**
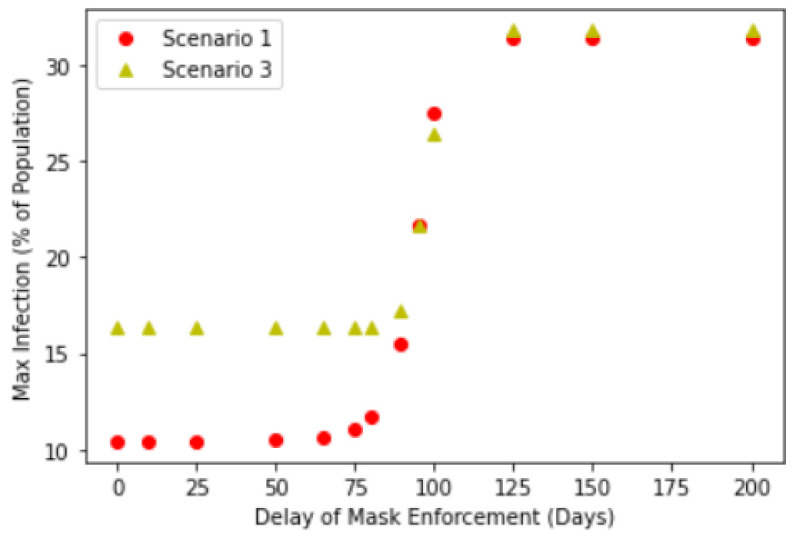
Maximum infection against delay in mask enforcement for Scenario 2: SEIR with full compliance and Scenario 3: SEIRS with time-limited immunity.

We further investigate this relationship by explicitly considering how the delay in mask enforcement will impact the corresponding Maximum Infected Values. Figure 4 shows the results of different enforcement delay values and their corresponding maximum infected values. Here, we considered 3 scenarios described in the introduction.

Figure 4 suggests a transition from low maximum infection of about 15–16% to a high maximum infection of about 31%, with the transition occurring at between a delay of 80 to 100 days. This transition manifests itself as a point of inflection of the graphs in Figure 4, occurring at about 100 days of delay. Interestingly, this suggests that delaying public masking enforcement for greater than about 100 days results in the significant reduction in the effectiveness of public masking in controlling the maximum infection numbers. This is because after 100 days, most of the Exposed compartment has already been been infected, therefore this will no longer contribute to any further increase to the Infection compartment. Relating this to Singapore’s context, compulsory mask wearing was introduced 89 days after the first case, hence this suggests that the introduction of compulsory mask wearing was timely to control the infection.

Crucially, one should note that the point of inflection appears to be independent of the choice of our scenarios and in all three cases, the transition takes place at about the same 80 to 100 days of delay. The peak infection beyond 100 days of delay also yield similar results in all three cases. One would naively anticipate that the noncompliance in Scenario 2 resulting in a larger infection rate and the backflow of Recovered to Susceptible compartment after 90 days of time-limited immunity would have contributed positively to the Maximum Infected Values. While this is indeed the case for early intervention, beyond the transition point, the Infected compartment is already on track in reaching its maximum (see Figure 3d), the effectiveness of any intervention to change its trajectory and reduce the maximum is greatly reduced. This suggests the importance of early intervention by policy makers and governments, where this potential window to take action is about 3 months based on our analysis. However, we stress this does not mean that public masking is completely ineffective after the transition point, but rather that its initial effectiveness is significantly reduced beyond this point, when studying the effects of compulsory mask wearing in isolation.

**Figure 5:**
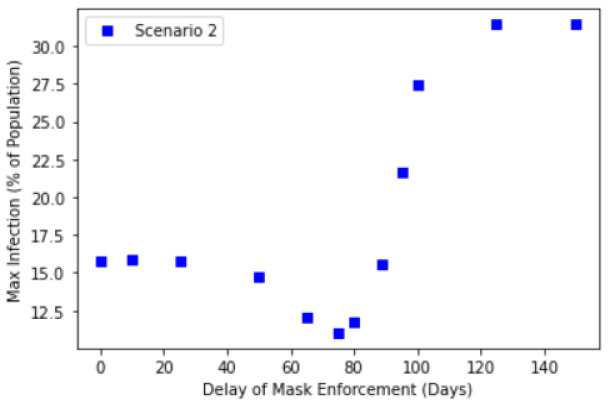
Maximum infection against delay in mask enforcement for Scenario 2: SEIR with gradual noncompliance.

We further note that in Scenario 2 (see Figure 5), the results suggest that earlier enforcement of mask wearing leads to higher than expected Maximum Infected Values (compared to Scenario 1). This is apparent for delays in mask enforcement under approximately 50 days. It is likely that for such cases of early enforcement, the Susceptible population remains very high throughout. Consequently, when the agents begin to flout the rules after $t_{1}=154$ days, the combination of both larger Susceptible compartment pool and higher $\beta_{nc}$ results in a greater amount of infection. In other words, the susceptible population must be sufficiently reduced before the implementation of the compulsory mask wearing for it to be effective in reducing the Maximum Infected Value. In practice, though not considered in this work, the susceptible population may be reduced or removed through other means, e.g., social distancing or lockdown.

**Figure 6:**
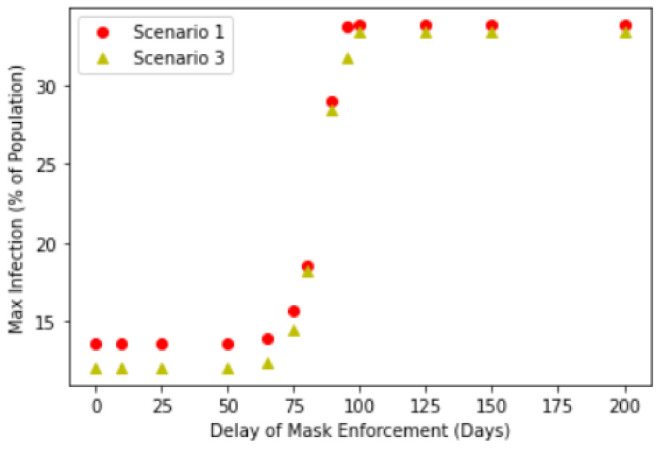
Maximum infection against delay in mask enforcement for Scenario 2: SEIR with full compliance and Scenario 3: SEIRS with time-limited immunity for the B117 variant case.

**Figure 7:**
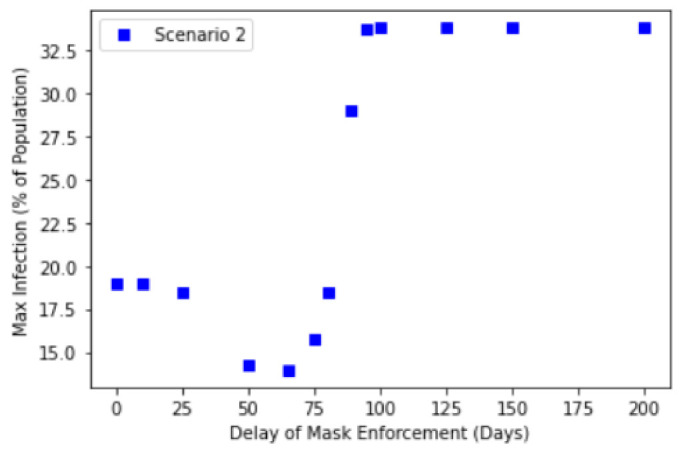
Maximum infection against delay in mask enforcement for Scenario 2: SEIR with gradual noncompliance for the B117 variant case.

At the time the initial study was conducted, we had modelled the COVID-19 situation in Singapore and its response to the initial strain of virus that was first detected at the start of 2020. However, the world is currently facing the second wave of infection. New strains, particularly the B117 variant, have been detected in many countries, including Singapore [23]. B117 strained virus has acquired the D614G spike protein mutation, reportedly increasing its infectivity, with little difference to its lethality [24]. According to studies done in the UK, the $R_{0}$ value of B117 has increased by up to 0.7 [25]. To investigate the impact of delay in public mask enforcement on controlling the Maximum Infected Numbers due to the B117 variant, we modelled a hypothetical scenario. In this scenario, the first case of COVID-19 detected in Singapore is of the B117 variant. We ran our simulation of the 3 scenarios described in the preceding sections with a new value of $R_{0}$ reflecting that of B117. The resulting plots are shown in Figure 6 and Figure 7.

### 6.3. Related Studies

Juxtaposing our study against other similar studies that implement mathematical modelling on ‘what-if’ scenario based analysis, Eikenberry et al. [3] implemented a compartmentalized models for their simulations, and concluded that if the population remains unmasked until mask enforcement after some discrete delay, and that the level of adoption is fixed, the delay initially had little impact on the hospitalized fraction or deaths, but states that a ‘point of no return’ can be crossed. While this study yields similar results to ours, it is contextualised in the US states of New York and Washington, whilst ours was conducted in the context of Singapore. Given the differences in epidemiological parameters, size and characteristics of these cities and Singapore, our study adds value in being a more accurate representation of smaller city states. Furthermore, this study did not take into consideration newer, more infectious strains of the virus such as the B117 variant and time-limited immunity, which we have considered in our study.

Another study done by Tatapudi et al. [26] on the impact assessment of various containment measures, including public masking, contact tracing and stay-home orders revealed that masking was indeed effective in lowering the maximum infected value. The simulation was carried out using Agent-Based modelling, where individual agents were generated from the census data from Florida. This study, like many others mentioned throughout this paper, did not explicitly study the impact of the delay in enforcement of public masking. However, it adds validity to the effectiveness of face masks in controlling the spread of infection, by means of a very different mathematical model.

### 6.4. Limitations of the Results

We caution against *exact* quantitative predictions of the development of pandemic that are dependent on a multitude of factors other than universal mask wearing that we have considered here. What we have considered here is the *what-if* analysis of the effects of implementation of compulsory mask wearing on the dynamics of the pandemic. While the parameters chosen were based on estimates in Singapore’s context, one should note that at the time of this writing, these estimates may change as we continue to deepen our understanding of the virus spread.

Instead, this work should be interpreted as a numerical representation of the possible outcomes of delaying mask enforcement as a basis for the discussion of the general trends. We further note that, at the time of writing, the actual real-world figures for Maximum Infected Values fall below the ones discussed in this paper. This is due to the fact that we have only examined the policy of compulsory mask wearing in isolation, with minimum consideration of other factors. We expect that any countries or territories that are actively fighting the virus spread to implement an array of varying measures, all of which would collectively reduce the overall spread of the virus.

While we acknowledge the limitations due to the rapidly changing nature of the ongoing pandemic and the presence of many contributing factors, some which are difficult to quantify, we cannot resort to waiting for sufficient data to present itself before drawing concrete solutions, as warned by Cheong and Jones [27], if we are to reduce its impact.

Analysing the results, as we may intuitively anticipate, given the increased infectivity of the new strain, the Maximum Infected Numbers obtained were of a slightly greater magnitude compared to the original model. Surprisingly, we observe the same consistent trend across all the 6 scenarios, comprising a point of inflection beyond which, the effectiveness of public masking is significantly reduced. The key difference to note is that the point of inflection occurs for a smaller value of delay in enforcement, approximately 80 days as opposed to the 100 days of our initial model. Returning to Singapore’s context, had the first case of COVID19 in Singapore been infected by the B117 variant, the delay of 89 days before public masking was enforced would have been costly.

## 7. Concluding Remarks

At present, compulsory mask wearing has been widely accepted as a means of controlling COVID-19 infection by reducing the infection rate through aerosol means. With this study, we hope to shed some light on whether the delay in enforcement of compulsory mask wearing will have detrimental effects on infection control when studied in isolation. Based on our results, it appears that a delay of 100 days and above would result in a transition where enforcement of public masking alone would result in little effect on the Maximum Infected Value. We considered 3 scenarios over 2 varied mathematical models. This result is consistent regardless of the level of compliance of the population, or the presence of time-limited immunity. Yet, if implemented early, our model shows that the Maximum Infected Values can be kept relatively low and under control, even after accounting for time-limited immunity and some level of noncompliance.

We would like to point out that several studies on the effectiveness of community use of face masks. In one study carried out in the United States [28], the authors compared the percentage change in daily case rates between states enforcing public masking and the states that do not. The results demonstrated that masking was indeed effective in reducing the number of infected cases daily. In their study, the states that enforced public masking did so between 8 April to 15 May 2020, approximately 78 days after the first case was detected in the US. Another study discussed the effectiveness of public masking by comparing countries that enforced public masking (Japan and Thailand) and countries that did not (Spain, Italy, UK, Germany and France) within 30 days after the first case was detected. The result of this study demonstrated that countries in the mask-wearing group had significantly better outcomes in containing the community spread of the virus [29]. These results therefore provide a basis to support our modelling and the conclusion that we have derived as a result of this study.
